# Opportunities for the development of drowning interventions in West Bengal, India: a review of policy and government programs

**DOI:** 10.1186/s12889-020-08868-2

**Published:** 2020-05-15

**Authors:** M. Gupta, A. B. Zwi, J. Jagnoor

**Affiliations:** 1 The George Institute for Global Health, University of New South Wales, Level 5/1 King St, Newtown, NSW 2042 Australia; 2 Health, Rights and Development (HEARD@UNSW), School of Social Sciences, Faculty of Arts and Social Sciences, UNSW Australia, Morven Brown Building, Kensington, 2052 Australia; 3 Injury Division, The George Institute for Global Health, New Delhi, 110025 India

**Keywords:** Drowning, Asphyxia, Wounds and injuries, Policy making, India, Safety, Government programs, Implementation science

## Abstract

**Background:**

Four million people living in the Indian Sundarbans region in the state of West Bengal face a particularly high risk of drowning due to rurality, presence of open water, lack of accessible health systems and poor infrastructure. Although the World Health Organization has identified several interventions that may prevent drowning in rural low-and middle-income country contexts, none are currently implemented in this region. This study aims to conduct contextual policy analysis for the development of a drowning program. Implementation of a drowning program should consider leveraging existing structures and resources, as interventions that build on policy targets or government programs are more likely to be sustainable and scalable.

**Methods:**

A detailed content review of national and state policy (West Bengal) was conducted to identify policy principles and/or specific government programs that may be leveraged for drowning interventions. The enablers and barriers of these programs as well as their implementation reach were assessed through a systematic literature review. Identified policies and programs were also assessed to understand how they catered for underserved groups and their implications for equity.

**Results:**

Three programs were identified that may be leveraged for the implementation of drowning interventions such as supervised childcare, provision of home-based barriers, swim and rescue skills training and community first responder training: the Integrated Child Development Scheme (ICDS), Self-Help Group (SHG) and Accredited Social Health Activist (ASHA) programs. All three had high coverage in West Bengal and considered underserved groups such as women and rural populations. Possible barriers to using these programs were poor government monitoring, inadequate resource provision and overburdening of community-based workers.

**Conclusions:**

This is the first systematic analysis of both policy content and execution of government programs to provide comprehensive insights into possible implementation strategies for a health intervention, in this case drowning. Programs targeting specific health outcomes should consider interventions outside of the health sector that address social determinants of health. This may enable the program to better align with relevant government agendas and increase sustainability.

## Background

Mortality from drowning presents a significant global burden, causing 320,000 deaths every year [1]. Drowning is defined as respiratory impairment resulting from submersion in liquid that may result in death or morbidity [2]. An estimated 90% of drowning events occur in low-and middle-income countries (LMICs) due to conditions such as poor infrastructure, poor regulation of water bodies and low awareness of water risks and swimming skills. Children aged 1–9 years old are at particular risk of drowning, especially in rural, coastal contexts where they are exposed to open water [3–5]. Drowning is in the top 5 causes of death for children aged 1–14 years old in 48 countries [1]. Drowning disproportionately affects underserved populations, especially those from lower socio-economic backgrounds in rural areas.

An estimated 62,000 drowning deaths occur in India each year, where it is the largest cause of child death by injury [6]. Despite this large burden, drowning is neglected as a public health issue. In particular, people living in the forested Sundarbans region in the state of West Bengal are exposed to a greater risk of drowning than many regions of India. It is remote and suffers from poor road and protective infrastructure such as fencing, unregulated open water, lack of safety awareness and inadequate health systems [7, 8]. The 4 million people living here are from lower socio-economic backgrounds than the rest of the state [9]. The remote locations of drowning events lead to underreporting to hospitals and police stations, and hence there is a lack of data on drowning [10, 11]. Climate change is increasing the frequency and intensity of flooding, exposing these populations to more dangerous conditions [7, 12, 13].

A drowning mortality survey in the Sundarbans (paper in preparation) found high rates of drowning in children aged 1–9 years old, at 244 deaths per 100,000 in children aged 1–4 years and 39 deaths per 100,000 in children aged 5–9 years. This is likely the leading cause of death for children in this region. Drowning interventions have not been implemented in the Sundarbans despite the risks. This paper presents the first component of formative research to develop a drowning intervention. The Steps for Quality Intervention Development (6SQuID) framework outlines the steps required for developing effective public health interventions [14]. According to this framework, once a health issue is identified, the micro, meso and macro context must be analysed to understand how the health intervention can be delivered [14–16]. In light of the Sustainable Development Goals (SDGs), the design of interventions should also consider issues of equity by analysing who is likely to benefit from, or be neglected by, an intervention [17, 18]. This is particularly relevant in the Sundarbans which is home to higher proportions of underserved groups than other parts of the state [7, 8, 18–20]. Drowning is more likely to affect these marginalised populations due to poorer living conditions, including lower access to piped water and less protection from open water sources [11, 21].

The World Health Organization (WHO) recommends four evidence-informed community-based interventions to reduce drowning. These address known risk factors, such as inadequate child supervision, poor post-drowning first response and proximity of water to homes [3]. Based on existing studies, expert recommendations and national and global data sources, these were the installation of barriers controlling access to water (such as playpens and fencing), the provision of supervision for pre-school aged children with capable child care, and provision of basic swimming and rescue skills along with training adult bystanders in safe rescue and resuscitation [3]. Interventions are more likely to be sustainable if they leverage existing structures and resources, and align with internal frames, goals and targets of the government [22–24]. Our analysis determined which public policies and social interventions would support the recommended WHO drowning prevention interventions.

This paper examines how national and state policy, policy targets and government programs in West Bengal, India relate (or could be related) to drowning interventions. We identified which policies and programs could be leveraged to implement community-based drowning interventions. We also evaluated how drowning-related government programs were being implemented in West Bengal, and how well they incorporated equity considerations. Our objectives were to: [1] identify conceptualisation and perceptions of drowning within Indian policy [2]; identify policies and programs that relate to WHO drowning interventions [3]; understand the reach and implementation of relevant government programs identified in policy and [4]; identify barriers, enablers and equity considerations to the implementation of these government programs.

## Methods

A systematic review of existing policy and literature was conducted to develop a comprehensive overview of the macro context in which a drowning intervention in the Sundarbans would operate. Policies and programs that may be leveraged for drowning interventions were identified. See Table 1 for inclusion criteria for data sources.

**Table 1:** Inclusion Criteria for data sources

Data source	Inclusion Criteria
Government policy documents	• Currently implementable as of July 2019 • Implementable in West Bengal • Discussion of principles, goals or programs that relate to drowning and/or WHO-recommended drowning reduction interventions
Grey literature	• Published from 1999 to 2019 • Discussion or evaluation of policy or programs in West Bengal that relate to drowning and/or WHO-recommended drowning reduction programs • If evaluation of a government program, GRADE score of Very Low was excluded
Peer-reviewed articles	• Published from 1999 to 2019 • Discussion or evaluation of policy or programs in West Bengal that relate to drowning and/or WHO-recommended drowning reduction programs • If evaluation of a government program, GRADE score of Very Low was excluded

For this analysis, policies were defined as any government documents that outlined principles, rules or guidelines. Programs were defined as implementable activities run and/or funded by the government.

### Data sources

Policy documents, peer-reviewed literature and grey literature sources were systematically sourced. Table 1 outlines the inclusion criteria for all sources.

#### Policy documents

The Sundarbans is located in the state of West Bengal and comes under the jurisdiction of both national Indian policy and West Bengal state policy. A thorough and structured review of relevant national and state-level websites was conducted to identify policies and programs that may be relevant to drowning interventions (Additional file 1).

A full text document review was conducted for all policy documents found. All policies were written in English. Only policies that were implementable in West Bengal as of July 2019 when this review was conducted were considered for analysis.

#### Other sources

After the identification of policies and programs that may be related to drowning, searches were conducted in both peer-reviewed and grey literature databases to gather literature on the implementation of these programs in West Bengal. These were then systematically screened by title and abstract, and remaining full texts analysed. See Additional files 2 and 3 for full search strategies, including list of databases and search terms.

Papers providing evaluations of government programs were assessed on the GRADE process, and articles rated ‘Very Low’ were removed [25]. Only literature analysing data from 1999 to July 2019 were considered.

### Analysis

For each policy identified, content analysis by the primary investigator identified [1] how drowning was conceptualised (including underlying assumptions, perceptions, definitions) [2]; policy and program relationships with WHO drowning reduction interventions; and [3] government targets and programs relating to child health, education or wellbeing.

Documents that did not discuss any policies or programs relating to drowning were excluded. Documents that discussed physical safety either in water or from water, such as relating to fencing around water bodies, were included.

To assess considerations of equity in policy and program design, policies that exhibited linkages to drowning interventions were analysed using the PROGRESS-PLUS framework [26] and a modified Equiframe framework [27]. The PROGRESS-PLUS framework identified which underserved groups were acknowledged. The Equiframe framework was modified to be applicable to non-health sectors including education and rural development, by removing health-specific criteria and language. This was used to analyse how well policies catered to the rights of underserved groups in rural contexts (See Additional file 4 for Equiframe Framework and modifications made). Analyses from these frameworks allowed us to build an understanding of the treatment of underserved populations in drowning-related policy and programs.

To make judgements on the enablers and barriers to government program implementation in West Bengal, peer-reviewed and grey literature were synthesised through a narrative approach [28].

## Results

### Document search results

A total of 18 policy documents were identified and 48 peer reviewed articles were included for the narrative evaluation of drowning-related government programs. See Additional file 5 for PRISMA flow diagram of search results and Additional file 6 for full list of policy documents included in analysis.

### Drowning and drowning reduction in policy

The policy review found very limited acknowledgement of drowning in any policy aside from some mention of drowning in disaster-related policy. The *National Disaster Management Plan 2016* acknowledged drowning as a cause of death during disasters. However, no specific mechanisms to avoid drowning were discussed. No other policy document specifically mentioned drowning (or physical water safety).

Health-related policy, including the *National Health Policy 2017*, showed greater focus on non-communicable diseases and included only minor reference to injuries. Policy related to the repair and management of water bodies, such as the *National Water Policy 2012* and the *Scheme on Repair, Renovation and Restoration (RRR) of Water Bodies under PMKSY (HKKP) 2017*, did not consider physical safety around water bodies. Like national-level policy, state-level policies from West Bengal did not mention drowning or physical water safety.

### Policy linkages to drowning interventions

**Fig. 1:**
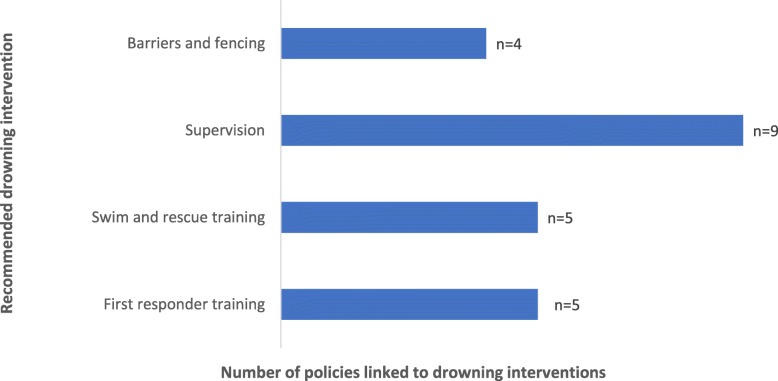
Policy linkages to drowning interventions

The review identified which policies described general principles or specific programs that related to the four drowning interventions recommended by WHO. Figure 1 shows how many policies mapped against each of the four recommended interventions followed by Table 2 which highlighted which programs were applicable. Policies that included principles relating to drowning reduction interventions did not necessarily specify programs to address these.

**Table 2:** Programs that may contribute to drowning interventions

Relevant government program identified in policy	Potential contribution to recommended drowning interventions			
	*Barriers and fencing*	*Supervised safe spaces*	*Swim and rescue training*	*First responder training*
Integrated Child Development Scheme (ICDS)	No	Yes	No	No
Mahatma Gandhi National Rural Employment Guarantee Act (MNREGA) 2005	No	Yes	No	No
Rajiv Gandhi National Crèche Scheme for the Children of Working Mothers 1994	No	Yes	No	No
National Bank for Agriculture and Rural Development (NABARD) Self-Help Group Bank Linkage Program	Yes	Yes	Yes	Yes
Accredited Social Health Activist (ASHA) program	Yes	Yes	Yes	Yes
Village Health, Sanitation and Nutrition Committees (VHSNC)	Yes	Yes	Yes	Yes

Table 2 summarises the government programs identified in relation to each WHO intervention. These programs were currently implemented by the National or West Bengal Government in the Sundarbans region as of July 2019.

The discussion below reflects on both the policies and programs that were identified that may be of value in taking forward specific drowning interventions.

#### Barriers and fencing

A few policies stated principles that aligned with the provision of barriers (Fig. 1). For example, the *National Policy for Children 2013* acknowledged that all children have the right to a safe, secure and protective environment, including at home. The *National Disaster Management Plan 2016* stated that dams and reservoirs should be enhanced for safety. However, the mechanisms through which safe environments should be ensured were not clearly defined, and no government programs had been introduced that aimed to provide physical protection from water bodies.

#### Supervised safe spaces

The provision of supervised safe spaces for children was the most supported drowning-related intervention in policy. Education policy, such as the *Early Childhood Care and Education Policy 2013* and *National Policy on Education 1992* both stated that children have the right to free early childhood education up to the age of 6 years old, provided through supervised crèche-based programs.

Three programs that provide crèche-based supervision were identified. Firstly, the Indian Government introduced and implemented the *Integrated Child Development Scheme (ICDS)*. The ICDS sets up local Anganwadi centres, where children are provided basic early childhood education and nutritionally-sufficient meals by locally trained women. West Bengal also launched *Shishu Aloy* in 2015, which aimed to transform some Anganwadi centres into enhanced early childhood learning centres with a structured pre-school curriculum.

A second program identified was the *Mahatma Gandhi National Rural Employment Guarantee Act (MNREGA) 2005*, which guaranteed 100 days of paid work to rural workers in the country. This program provisioned that a crèche service must be provided in any workplace where more than five children aged under 6 years old attend with their mothers.

Lastly, the *Rajiv Gandhi National Crèche Scheme for the Children of Working Mothers 1994* aimed to provide day-care facilities for children aged 6 months to 6 years in both urban and rural community-based settings.

#### Swim and rescue training

There was no policy requirement found for children to learn swimming skills. Education policy, such as the *National Policy on Education 1992,* necessitated the provision of physical activity classes in schools with the appropriate infrastructure (Fig. 1). However, policy did not identify swimming as a requirement within this. No specific swim and rescue training programs have been introduced by government.

#### First responder training

Policy focussed on building the capacity of communities to manage their own health and disaster response. From this view, the principle of providing first responder training was well supported. The *National Health Policy 2017* aimed to increase the number of ‘health volunteers’ in communities who may act as first responders for health concerns. The *National Disaster Management Plan 2016* and *West Bengal State Disaster Management Policy and Framework* also required community members, especially local leaders, to be trained in first response to respond to accidents and natural disasters. However, no specific government programs to provide First Responder training had been introduced in West Bengal based on these national directions.

#### Comprehensive programs

However, three valuable programs that may play facilitative or implementation roles across all four drowning interventions were also identified.

Firstly, Self-Help Group (SHG) programs, including the *National Bank for Agriculture and Rural Development (NABARD) Self-Help Group Bank Linkage Program* and the *National Rural Livelihoods Mission*, were identified as possible facilitators for program delivery. These programs formed support groups in villages for the provision of loans, mostly with women. The *National Policy for the Empowerment of Women 2001* had identified SHGs as a mode through which social and economic development programs can be implemented in communities.

Secondly, the *Accredited Social Health Activist (ASHA)* program was mandated in the *National Health Mission 2013* policy. The ASHA program trained and deployed community-based maternal and child health workers to act as the interface between the community and public health system. They were tasked with educating and mobilising communities on health issues through household visits and community meetings.

Thirdly, the *National Health Mission 2013* detailed the establishment *of Village Health, Sanitation and Nutrition Committees (VHSNCs)*, which were sub-committees under the local government (Gram Panchayats) tasked with overseeing programs related to health and its social determinants.

### Equity considerations in policy

**Fig. 2:**
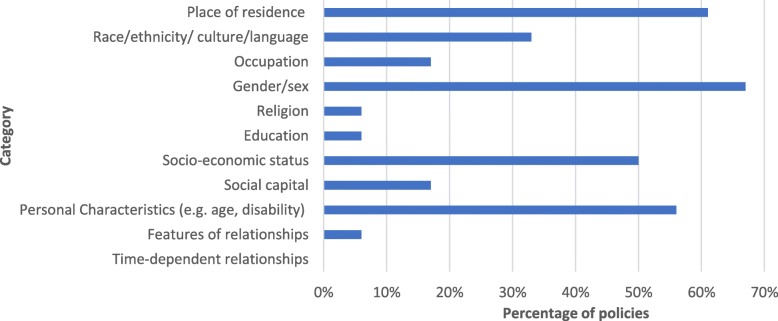
PROGRESS-Plus analysis

Particular barriers faced by women were mentioned in 67% of the policy documents (See Fig. 2). Place of residence was acknowledged in 61% of policies and socio-economic status in 50%. Social capital, educational background, occupation and religion were rarely discussed. Only two policies acknowledged more than half of the PROGRESS-PLUS groups: the *National Health Policy 2017* and the *National Policy for the Empowerment of Women 2001* (Fig. 2).

Based on the Equiframe Framework, 78% (*n* = 14) of policies acknowledged that programs should be tailored to meet the needs of underserved groups, and 72% (*n* = 13) recognised that underserved groups were capable and had a right to be involved in decision making. 61% (*n* = 11) of policies also recognised explicitly that underserved groups were productive contributors to society, and 56% (*n* = 10) supported underserved groups’ physical, economic, and information access to services. However, few policies aimed to protect the privacy of underserved groups’ information, ensured programs were tailored to the person’s characteristics, or acknowledged the role of family in the impact of services. See Table 3 for the number of policies that addressed each Equiframe domain.

**Table 3:** Equiframe analysis

Domain	Number of policies
Does the policy support the rights of underserved groups with equal opportunity in receiving services?	9
Does the policy support the rights of underserved groups with individually tailored services to meet their needs and choices?	14
Does the policy indicate how underserved groups may qualify for specific benefits relevant to them?	5
Does the policy recognize the capabilities existing within underserved groups?	13
Does the policy support the right of underserved groups to participate in the decisions that affect their lives and enhance their empowerment?	10
Are underserved groups protected from harm during their interaction with health and related systems?	5
Does the policy support the right of underserved groups to be free from unwarranted physical or other confinement?	5
Does the policy support the right of underserved groups to consent, refuse to consent, withdraw consent, or otherwise control or exercise choice or control over what happens to him or her?	6
Does the policy address the need for information regarding underserved groups to be kept private and confidential?	1
Does the policy recognize that underserved groups can be productive contributors to society?	11
Does the policy recognize the value of the family members of underserved groups in addressing health and safety needs?	6
Does the policy recognize that individual members of underserved groups may have an impact on the family members, requiring additional support from health or other related services?	2
Does the policy ensure that services respond to the beliefs, values, gender, interpersonal styles, attitudes, cultural, ethnic, or linguistic aspects of the person?	5
Does the policy specify to whom, and for what, services providers are accountable?	9
Does the policy support underserved groups’ physical, economic, and information access to services?	10

Policies that linked to supervision-based interventions were more likely to protect the rights of underserved groups. For example, both the *Early Childhood Care and Education Policy 2013* and the *National Population Policy 2016* catered to 80% (*n* = 12) of the applicable Equiframe criteria, and *MGNREGA* and catered to 77% (*n* = 10) of the applicable criteria. However, water and disaster-related policies, which often considered First Responder training and barriers and fencing, included few or no equity considerations. Health-related policy reflected only half of the Equiframe criteria.

### Implementation of programs with linkages to drowning reduction

As discussed above, a range of government programs were identified in policy that may be leveraged in support for drowning reduction. The following reviews what is known about the implementation of these programs in West Bengal based on a systematic review of the peer-reviewed literature.

#### Integrated child development scheme (ICDS)

The Integrated Child Development Scheme (ICDS) is delivered through community-based Anganwadi centres. A trained female Anganwadi worker and assistant are responsible for running the centre 4 hours per day from 7 to 11 am. They are tasked with providing early childhood education (ECE) and nutritionally-sufficient meals [29, 30]. In West Bengal, the lowest tier of local government, the Gram Panchayat, is responsible for implementing the ICDS program [31]. Anganwadi workers are paid a stipend and are considered volunteers [32].

Overall, 88% of villages in West Bengal have access to an Anganwadi centre, compared to 45% nationally [33]. However, cross-sectional survey data in two districts found that 27% of centres documented as operational were not running [34]. The true coverage of Anganwadi centres is likely to be lower than reported.

Implementation of ECE in Anganwadi centres is variable across the state, with only 60–85% of centres in each district providing this service [30, 35–37]. A cross-sectional study found that where provided, the duration of ECE activities averaged at only 60 min, below the 120 min prescribed in policy. In addition, only 10% of centres had sufficient floor space for indoor activities, 33% did not have any materials for activities, and none ran activities that were age appropriate. One study found that only 33% of children enrolled at the centre remained to participate in ECE [38].

A key barrier to implementing ECE in Anganwadi centres was the lack of an educational curriculum against which the services could be assessed. In addition, Anganwadi workers had limited resources and time to run ECE activities between their other responsibilities, and viewed the service as effortful [32, 35, 39]. Anganwadi centres may require additional resources in order to provide these services [40].

The ICDS program suffers from poor supervision and administration. Funding is often inadequate for rent, food and materials, and corrupt practices in higher levels of administration have occurred [32]. Furthermore, there is limited capacity to provide ongoing training, supervision and support of Anganwadi workers in managing on-ground implementation [41]. Although many Indian policies highlight ECE as a right, little emphasis is placed on ECE activities by supervisors [35].

#### Shishu Aloy

In 2015, the West Bengal government launched the *Shishu Aloy* program to set up enhanced Anganwadi centres that focused on early childhood education with a structured curriculum. However, there is no data available on this program’s coverage and implementation success.

#### Self-help group (SHG) programs

The *National Bank for Agriculture and Rural Development (NABARD) Self-help Group Bank Linkage program* and the *National Rural Livelihoods Mission* are two programs centred on supporting mainly women through support groups. Although these two programs are independent, the basic models are similar [42, 43]. Village-based self-help groups are formed by partnering NGOs and banks and linked to a financial institution for the provision of loans [44]. They also become involved in the provision of non-public services, such as providing training on agricultural techniques and providing childcare and healthcare [45, 46].

West Bengal has one of the greatest reaches of SHG programs in the country, with 51–75% of rural households being covered [47–50]. SHGs in West Bengal have been found to be effective in increasing women’s income, providing better access to credit, reducing reliance on local money-lenders, reducing physical labour for members, increasing members’ autonomy, increasing employment, improving access to health information and services, reducing rural poverty and empowering women with decision making power in their families and communities [19, 47, 51–59].

Nevertheless, challenges are evident. Firstly, there is often inadequate mentorship from the linked banks and NGOs for navigating the financial system [51, 52, 60, 61]. This is attributable to the absence of a clear chain of authority and accountability in funding structures [52, 62]. Secondly, women still bear the brunt of domestic work in rural communities, which may inhibit their ability to be involved in community activities [53, 63]. Operational issues have also been reported, where regularity of meetings and coordination between members may be poor [51].

The benefits and function of SHGs varies across religious and caste groups. Economic and political benefits have been found to be higher for upper caste Hindus than scheduled caste Hindus or Muslims, as they start with greater social mobility, higher incomes and better access to markets and technologies [19, 51]. Muslim women in particular see fewer improvements in business profits or health outcomes compared to Hindu women, possibly due to greater restrictions on women’s roles and mobility [64].

#### Village health, sanitation and nutrition committees

The *National Rural Health Mission* set up *Village Health, Sanitation and Nutrition Committees* at the village level. These are sub-committees to the Gram Panchayat that coordinate collective actions on issues related to health and its social determinants. This includes overseeing the functioning of Anganwadi Centres and facilitating the work of front line health workers in the community [65]. 29.4% of villages in West Bengal have VHSNCs, similar to the national average [66, 67]. However, there is no information on their effectiveness and involvement in on-ground implementation in rural West Bengal.

#### Rajiv Gandhi National Crèche Scheme for working mothers

This program seeks to open crèche services for working mothers within communities. A key difference between these crèches and Anganwadi centres is that they run for 7.5 h each day, rather than half days. Currently there are 1636 crèches operating in West Bengal under this scheme, serving 40,900 children. However, the coverage of centres is low due to insufficient funding for these centres, and delays in payments to implementing agencies [68].

#### Mahatma Gandhi National Rural Employment Guarantee act (MGNREGA)

In West Bengal, 33% of the labour employed through MGNREGA are women [59]. However, evidence from surveys conducted in West Bengal indicate that few workplaces have functioning childcare facilities available for use, and women are not aware of their right to request these services. Gram Panchayats also do not view the provision of childcare services a priority and are not taking actions to ensure set up [59, 69, 70].

#### Accredited social health activist (ASHA) program

The *Accredited Social Health Activist (ASHA)* program is part of the *National Health Mission* strategy. In 2016, there were 47,204 ASHAs employed in West Bengal at a population density of 1 per 1317 population. This is 77% of the target of 1 per 1000 population [71]. ASHAs in West Bengal perform above average compared to the other states, showing higher members of visitations to households and greater involvement in sanitation education and toilet construction [72]. However, ASHAs in West Bengal are rarely fulfilling maternal support duties, with 7% of ASHAs escorting women to health centres at the time of delivery, and only 14% visiting on the first day of birth. One study in the Howrah District of West Bengal found that half of ASHA workers were overburdened. In addition, Muslim ASHAs showed lower performance due to lower educational attainment and less support from Anganwadi workers [73].

## Discussion

This paper aimed to develop a comprehensive understanding of the macro level policy context offering potential for drowning interventions. The results show a severe lack of awareness of drowning as an issue in policy. This is likely to result from a lack of available data on drowning and cultural beliefs that drownings are “accidents” [10, 11]. The majority of data collection systems in India such as the *Samples Registration System* and *District Level Household and Facility Survey* collect information on deaths caused by injury more broadly but not specifically drowning deaths. Drowning is specified in police data (called the *National Crime Records Bureau* data) and in Medical Death Certificate data which feeds into the *Vital Registration System*, but low reporting to police stations and hospitals leads to a vast underestimate. Deaths are unlikely to be reported due to the rurality of incidents and the high number of deaths at the place of drowning that do not require medical attention. In addition, there are few legal implications of drowning deaths for both families and police as they are largely accidental and involve children.

### Building on pre-existing government programs

The ICDS program, ASHA program and SHGs programs are all government programs that may be leveraged for drowning interventions. While the ICDS program may be optimised for child supervision, the ASHA and SHG programs have the flexibility to be leveraged across the range of drowning interventions.

#### Integrated child development scheme (ICDS)

The ICDS program stipulates providing 2 h of early childhood education activities for children aged 3–6 years old through Anganwadi centres. They have the potential to be used for structured supervision programs by extending ECE activity timings. Angwanwadi workers view themselves as respectable teachers and are motivated to contribute to initiatives that enhance child health outcomes [32]. They may also be used as a source of information on child deaths by drowning, as they already collect information on a range of child issues.

If Anganwadi centres are to be used for childcare services, additional factors must be considered. Firstly, Anganwadi workers are already heavily burdened in their duties and already struggle to provide the 2 hours of daily ECE activities mandated [32]. They are involved in a range of activities including community-level data collection on child issues and children’s meals. The workloads of Anganwadi workers would need to be re-assessed to ensure that childcare services can be sustainably provided.

Low participation in the Anganwadi childcare program suggests that parents may not see the value in ECE. Mothers have also reported low levels of trust in Anganwadi workers to provide quality and safe services [34, 74]. Community trust building exercises will be required to encourage mothers to send their children for childcare services offered.

Another consideration is the effect of caste and religion on service provision. Children from a different caste or religion to the Anganwadi worker are sometimes treated poorly. Muslim attendance at Anganwadi centres is also lower compared to other groups [75, 76]. The provision of childcare services in a diverse community must understand and take account of these dynamics.

Anganwadi program policy acknowledged gender norms and rurality as barriers to access. It is important that ICDS addresses the effects of gender on access, as it is largely mothers who bring children to Anganwadi centres [34, 77].

#### ASHAs

ASHA workers are heavily involved in the community and have close relationships with women, making them advantageous for intervention delivery. However, as with Anganwadi workers, a key consideration for the use of ASHA workers is that they are overburdened with many duties such as household visits, data collection, disseminating vaccination information and more. The total number of tasks ASHAs must perform has increased from six in 2005 to 28 in 2017 [78]. ASHA workers must also attend to women in labour at short notice. Hence, ASHAs may be involved in community mobilisation activities such as engaging communities in first responder training or barrier installation, but are unlikely to have the time to provide child supervision or swim training themselves.

Poor consideration of equity in health-related policy may also be problematic when ensuring any interventions delivered by ASHAs cater to different groups, especially as ASHAs from minority groups have been shown to have poorer performance [73]. To ensure equitable reach of programs, barriers faced by some ASHAs should be identified and addressed with differential strategies and support.

#### Self-help groups

Self-help groups could be leveraged in the implementation and support of any of the drowning prevention programs, given their high coverage of rural households and involvement in community-based social activities. They are also largely composed of women who are viewed culturally as the main carers of children. As self-help groups programs face less regulation and government-level constraints compared to the ASHA and ICDS programs, they may be simpler to engage in implementation.

### Government programs less feasible for drowning interventions

While the *Mahatma Gandhi National Rural Employment Guarantee Act (MGNREGA)* includes a provision to provide childcare services to working women, this scheme will not cover a large number of children. The participation rate of women in the formal workforce is low, particularly in rural areas [79]. Poor coverage of the *Rahul Ghandi Crèche Scheme for Working Mothers* and *Village Health, Sanitation and Nutrition Committees* also reduce its usefulness for the implementation of a region-wide drowning intervention.

### Practical considerations of leveraging existing government programs

While documented policy provides a window into government programs that may be leveraged for drowning interventions, it is essential to understand government decision makers’ perceptions towards drowning [23, 80]. To ascertain which of the above discussed government programs can be feasibly leveraged, government processes behind the implementation of these programs must be understood. Furthermore, interactions between government decision makers, implementers and community-based staff should be taken into account in intervention design. This ensures that appropriate stakeholders are engaged and implementation can occur with governmental cooperation [81, 82]. Further qualitative research with policy makers and government implementers will capture their perspectives.

Policies and programs from other sectors should be considered when designing programs to address health outcomes especially if they address the social determinants of health [83]. This broadens the scope for health researchers and planners to engage with different government sectors to consider a wider range of intervention designs. For example, the ICDS and Self-help Group programs come under the Ministry of Women and Child Development and are linked with the Ministry of Education, while the ASHA program sits under the state level Ministry of Health. The policies related to first responder training and barriers relate to water infrastructure and disaster-related ministries. Inter-sectoral collaboration is required to implement these drowning reduction interventions.

The challenges for inter-sectoral collaboration are well-noted and must be considered. In many LMIC contexts, district governments are the main implementers of cross-sectoral programs, but coordination platforms and systems to track progress, engage experts or manage resources across departments are absent [84, 85]. Competing priorities and a lack of incentives to coordinate may also prevent collaboration [86]. In order for intersectoral collaboration to occur, departments must set up regular communication systems, clarify incentives, targets and performance indicators, and empower strong leadership [85–88]. Bureaucratic resource systems also need to support collaboration, which requires buy-in from people within a range of departmental bureaucracies [89].

In addition to top-down changes, bottom up action is required to promote government accountability for implementation. Community awareness and education campaigns about drowning risks and responses should be integrated with intervention delivery, helping to raise public interest and accountability. Garnering community support may stimulate more pressure on local governments to meet implementation targets [90–92].

### Development of new programs based on policy principles

In addition to using existing government programs for the implementation of drowning reduction interventions, the development of new interventions that align with policy goals and principles may also be feasible. New programs that align with existing policy are more likely to be accepted by government and implemented once their efficacy has been established [23].

These results show that the provision of first responder training is highly supported in disaster risk reduction and climate change policy. The establishment of safe spaces for children is also acknowledged in children’s and education policy, supporting the implementation of barrier-based interventions. Hence, working with government stakeholders to develop new programs that cater to policy goals is an additional strategy for developing drowning reduction interventions.

### Limitations of the study

Firstly, not all state-level policy documents may have been available online to the public. While we made contact with state government offices for access to any other policy documents that may exist, they were non-responsive. To ensure we were basing analysis on all publicly-available data, we re-checked all websites after the analysis to ensure no policy documents were missed. Secondly, there were limited primary studies of government program implementation from the Sundarbans region, so the search was extended to all West Bengal. Some of the findings of implementation reach and success of government programs may not be applicable to implementation in the Sundarbans region. Further research is needed that conducts primary qualitative work with program implementers in this region to identify specific enablers and barriers for the region.

### Implications for future study

This study provides guidance on how the implementation of drowning programs in the Sundarbans region may leverage on government programs. However, implementation will involve the expertise and buy-in of both government and Sundarbans communities. To understand both policy and community-level perceptions and considerations of drowning program design and implementation, qualitative work with relevant stakeholders is required. Community participatory approaches will enable development of an acceptable and pragmatic model for the region, given its unique geography and low-resource setting [93, 94].

## Conclusions

Drowning is a major cause of child death by injury in India, particularly in the rural, coastal Sundarbans region in the state of West Bengal. However, no interventions are currently implemented in this region to address this issue, such as supervised childcare, home-based barriers, swim and rescue training or first responder training as recommended by World Health Organization. This paper identified government policy and programs that mapped against the recommended interventions and could be leveraged for the implementation of a sustainable drowning program. The analysis enabled the identification of ‘win-win’ solutions that not only addressed the drowning problem, but also enabled the government to meet its short and long term goals, including across different sectors, to enhance program sustainability and feasibility [95]. Drowning interventions that leverage and improve existing structures such as the ICDS program, ASHA program and SHG programs can be both beneficial in reducing drowning and improving the implementation of these schemes for government. New drowning interventions that cater to policy targets, such as those around upskilling communities in first response and providing safe spaces for children at home, also warrant consideration. As drowning disproportionately impacts children and marginalised, rural communities, addressing drowning will also play a key role in supporting West Bengal to meet its SDG targets, particularly those relating to under-5 mortality and reducing inequities [96].

## Supplementary information

**Additional file 1.** Appendix 1: Indian National and State Government searches for policy documents.

**Additional file 2.** Appendix 2: Peer-reviewed database search results.

**Additional file 3.** Appendix 3: Grey literature database search results.

**Additional file 4.** Appendix 4: Modified Equiframe Framework.

**Additional file 5 **Appendix 5: *PRISMA Flow Diagram.*

**Additional file 6.** Appendix 6: List of policy documents included in final analysis.

## Data Availability

Data sharing is not applicable to this article as no datasets were generated or analysed during the current study.

## Abbreviations

ASHA: Accredited Social Health Activist
ECCE: Early Childhood Care and Education
ECE: Early Childhood Education
ICDS: Integrated Child Development Scheme
LMICs: Low-and middle-income countries
MNREGA: Mahatma Gandhi National Rural Employment Guarantee Act 2005
NABARD: National Bank for Agriculture and Rural Development
NCW: National Commission for Women
NHRC: National Human Rights Commission
SHG: Self-Help Group
6SQuID: Steps for Quality Intervention Development
SDGs: Sustainable Development Goals
WHO: World Health Organization
VHSNCs: Village Health, Sanitation and Nutrition Committees
