# Supplementary material for: Opportunities for the development of drowning interventions in West Bengal, India: a review of policy and government programs
Source: BMC Public Health. 2020 May 15;20:704. doi: 10.1186/s12889-020-08868-2 (PMC7229618; doi:10.1186/s12889-020-08868-2)
Supplement: Supplementary file 1 — Additional file 1. Appendix 1: Indian National and State Government searches for policy documents. [file 12889_2020_8868_MOESM1_ESM.docx]

### Appendix 1: Indian National and State Government searches for policy documents

| Department | No. of policy documents obtained |
| --- | --- |
| National Commission for Women (NCW) | 0 |
| National Department of Education | 1 |
| National Human Rights Commission (NHRC) | 1 |
| National Institute of Hygiene and Public Health | 0 |
| National Ministry for Women and Child Development | 5 |
| National Ministry of Environment, Forest and Climate Change | 1 |
| National Ministry of Health & Family Welfare | 3 |
| National Ministry of Rural Development | 1 |
| National Ministry of Social Justice and Empowerment | 0 |
| National Ministry of Tribal Affairs | 1 |
| National Ministry of Water Resources | 4 |
| West Bengal State Fisheries Development Corporation, Ltd, Kolkata | 0 |
| West Bengal Institute of Health and Family Welfare | 0 |
| West Bengal State Department of Backward Classes Welfare | 0 |
| West Bengal State Department of Disaster Management and Civil Defence | 4 |
| West Bengal State Department of Environment | 1 |
| West Bengal State Department of Health and Family Welfare | 0 |
| West Bengal State Department of Irrigation and Waterways | 0 |
| West Bengal State Department of Mass Education Extension and Library Services | 0 |
| West Bengal State Department of Panchayat and Rural Development | 1 |
| West Bengal State Department of Public health Engineering | 0 |
| West Bengal State Department of School Education | 0 |
| West Bengal State Department of Tribal Development | 0 |
| West Bengal State Department of Water Resources Investigation and Development | 0 |
| West Bengal State Department of Women and Child Development and Social Welfare | 1 |
| Snowballed | 2 |
| Commonwealth Human Rights Initiative | 0 |
| TOTAL | **26** |
