# Supplementary material for: Opportunities for the development of drowning interventions in West Bengal, India: a review of policy and government programs
Source: BMC Public Health. 2020 May 15;20:704. doi: 10.1186/s12889-020-08868-2 (PMC7229618; doi:10.1186/s12889-020-08868-2)
Supplement: Supplementary file 3 — Additional file 3. Appendix 3: Grey literature database search results. [file 12889_2020_8868_MOESM3_ESM.docx]

### Appendix 3: Grey literature database search results

| Database | Search syntax | No. of results |
| --- | --- | --- |
| World Health Organization Library Database | (India* OR “West Bengal”) AND (policy OR policies OR scheme* OR program* OR intervention) AND (drown* OR “near-drowning” OR water* OR safe* OR educat* OR rural OR child* OR infant* OR swim* OR rescu* OR disaster* OR research OR submer* OR injur*) | 1 |
| United Nations Official Document System | West Bengal  Drown* | 0 |
| Google search | Drowning policy site:.in filetype:pdf | 0 |
| Human Rights Documents Online | India drown* | 220 |
