# Supplementary material for: Opportunities for the development of drowning interventions in West Bengal, India: a review of policy and government programs
Source: BMC Public Health. 2020 May 15;20:704. doi: 10.1186/s12889-020-08868-2 (PMC7229618; doi:10.1186/s12889-020-08868-2)
Supplement: Supplementary file 4 — Additional file 4. Appendix 4: Modified Equiframe Framework. [file 12889_2020_8868_MOESM4_ESM.docx]

### Appendix 4: Modified Equiframe Framework

| **Number** | **Question** |
| --- | --- |
| 1 | Does the policy support the rights of underserved groups with equal opportunity in receiving services? |
| 2 | Does the policy support the rights of underserved groups with individually tailored services to meet their needs and choices? |
| 3 | Does the policy indicate how underserved groups may qualify for specific benefits relevant to them? |
| 4 | Does the policy recognize the capabilities existing within underserved groups? |
| 5 | Does the policy support the right of underserved groups to participate in the decisions that affect their lives and enhance their empowerment? |
| 6 | Are underserved groups protected from harm during their interaction with health and related systems? |
| 7 | Does the policy support the right of underserved groups to be free from unwarranted physical or other confinement? |
| 8 | Does the policy support the right of underserved groups to consent, refuse to consent, withdraw consent, or otherwise control or exercise choice or control over what happens to him or her? |
| 9 | Does the policy address the need for information regarding underserved groups to be kept private and confidential? |
| 10 | Does the policy recognize that underserved groups can be productive contributors to society? |
| 11 | Does the policy recognize the value of the family members of underserved groups in addressing health and safety needs? |
| 12 | Does the policy recognize that individual members of underserved groups may have an impact on the family members, requiring additional support from health or other related services? |
| 13 | Does the policy ensure that services respond to the beliefs, values, gender, interpersonal styles, attitudes, cultural, ethnic, or linguistic aspects of the person? |
| 14 | Does the policy specify to whom, and for what, services providers are accountable? |
| 15 | Does the policy support underserved groups’ physical, economic, and information access to services? |

Modifications made:

1. “Vulnerable groups” was changed to “Underserved groups”
2. Questions pertaining to ‘Health services’ were reworded to ‘services’ only.
3. Questions that stated “Health needs” these were expanded to state “Health and safety needs.”
4. The following questions were removed from the original framework
   1. “Does the policy support assistance of vulnerable groups in accessing services from within a single provider systems (intra-agency) or more than one provider system (inter-agency) or more than one sector (intersectoral)?”
   2. “Does the policy promote the use of mainstream services by vulnerable groups?”
   3. “Does the policy support vulnerable groups in seeking primary, secondary and tertiary prevention of health conditions?”
   4. “Does the policy support the capacity building of health works and of the system that they work in addressing health needs of vulnerable groups?”
   5. “Vulnerable groups are assured of the quality of the clinically appropriate services.”
   6. “Does the policy support efficiency by providing structured way of matching health system resources with service demands in addressing the health needs of vulnerable groups?”
