# Supplementary material for: Opportunities for the development of drowning interventions in West Bengal, India: a review of policy and government programs
Source: BMC Public Health. 2020 May 15;20:704. doi: 10.1186/s12889-020-08868-2 (PMC7229618; doi:10.1186/s12889-020-08868-2)
Supplement: Supplementary file 5 — Additional file 5 Appendix 5: PRISMA Flow Diagram. [file 12889_2020_8868_MOESM5_ESM.docx]

**Appendix 5: PRISMA Flow Diagram**

Records identified through database searches

(n = 80,498)

Records identified through grey literature searches

(n = 221)

Policies identified and screened through Government websites

(n = 26)

Potentially relevant papers retrieved for examination of full text based on title and abstract review

Grey literature: (n = 2)

Peer-reviewed: (n = 320)

Papers included in final analysis

- Policy documents (n = 18)
- Peer-reviewed papers (n = 48)
- Grey literature (n = 0)

Papers excluded on full text review

No analysis or discussion of programs relating to drowning reduction: (n = 201)

Very Low GRADE score (n = 18)

Wrong location of analysis: (n = 52)

Data older than 1999: (n = 7)

Full text unavailable: (n = 4)

Number records after removal of duplicates

(n = 37,988)
