# Supplementary material for: Opportunities for the development of drowning interventions in West Bengal, India: a review of policy and government programs
Source: BMC Public Health. 2020 May 15;20:704. doi: 10.1186/s12889-020-08868-2 (PMC7229618; doi:10.1186/s12889-020-08868-2)
Supplement: Supplementary file 6 — Additional file 6. Appendix 6: List of policy documents included in final analysis. [file 12889_2020_8868_MOESM6_ESM.docx]

**Appendix 6: List of policy documents included in final analysis**

1. National Health Policy 2017
2. National Early Childhood Care and Education (ECCE) Policy 2013
3. Operational Guidelines for Mahatma Gandhi National Rural Employment Guarantee Act 2005
4. National Policy for the Empowerment of Women 2001
5. National Water Policy 2012
6. National Policy on Education 1992
7. National Action Plan and Monitoring Framework for Prevention and Control of NCDs 2013
8. National Nutrition Policy 1993
9. National Population Policy 2000
10. National Disaster Management Plan 2016
11. The National Policy for Children 2013
12. National Water Mission 2011
13. West Bengal State Action Plan on Climate Change 2012
14. Rajiv Gandhi National Crèche Scheme for the Children of Working Mothers 1994
15. Community Health Care Management Initiative 2013
16. Shishu Aloy, 2012
17. West Bengal State Disaster Management Policy and Framework 2012
18. National Health Mission 2013 (Manual for District-Level Functionaries)
