# Supplementary material for: Opportunities for the development of drowning interventions in West Bengal, India: a review of policy and government programs
Source: BMC Public Health. 2020 May 15;20:704. doi: 10.1186/s12889-020-08868-2 (PMC7229618; doi:10.1186/s12889-020-08868-2)
Supplement: Supplementary file 2 — Additional file 2. Appendix 2: Peer-reviewed database search results. [file 12889_2020_8868_MOESM2_ESM.docx]

### Appendix 2: Peer-reviewed database search results

| Database | Search Syntax | No. of results |
| --- | --- | --- |
| Medline | (India*.mp OR INDIA/ OR West Bengal.mp) AND (POLICY/ OR policies.mp OR scheme*.mp OR program*.mp OR intervention.mp) AND (drown*.mp OR near-drown*.mp OR water*.mp OR safe*.mp OR educat*.mp OR rural.mp OR child*.mp OR CHILD/ OR infant*.mp OR INFANT/ OR swim*.mp OR rescu*.mp OR disaster* OR RESEARCH/ OR submer*.mp OR injur*.mp) | 4670 |
| EMBA SE | (India*.mp OR INDIA/ OR West Bengal.mp) AND (POLICY/ OR policies.mp OR scheme*.mp OR program*.mp OR intervention.mp) AND (drown*.mp OR DROWNING/ OR near-drown*.mp OR water*.mp OR safe*.mp OR educat*.mp OR rural.mp OR child*.mp OR infant*.mp OR swim*.mp OR rescu*.mp OR disaster* OR DISASTER/ OR RESEARCH/ OR submer*.mp OR injur*.mp) | 8367 |
| Emcare | (India*.mp OR INDIA/ OR West Bengal.mp) AND (POLICY/ OR policies.mp OR scheme*.mp OR program*.mp OR intervention/) AND (drown*.mp OR water*.mp OR water/ OR safe*.mp OR educat*.mp OR rural.mp OR child*.mp OR infant*.mp OR swim*.mp OR rescu*.mp OR disaster* OR disaster/ OR research/ OR submer*.mp OR injur*.mp) | 5071 |
| Global Health | (India*.mp OR West Bengal/) AND (Policy/ OR policies.mp OR scheme*.mp OR program*.mp OR intervention.mp) AND (drown*.mp OR DROWNING/ OR near-drown*.mp OR water*.mp OR WATER/ OR safe*.mp OR educat*.mp OR rural.mp OR child*.mp OR CHILD/ OR infant*.mp OR INFANT/ OR swim*.mp OR rescu*.mp OR disaster* OR DISASTER/ OR RESEARCH/ OR submer*.mp OR injur*.mp) | 5848 |
| Scopus | TITLE-ABS-KEY ( ( india OR indian OR "West Bengal" ) AND ( policy OR policies OR scheme* OR program* OR intervention ) AND ( drown* OR "near-drowning" OR water* OR safe* OR educat* OR rural OR child* OR infant* OR swim* OR rescu* OR disaster* OR submer* OR injur* ) ) AND PUBYEAR > 1997 | 29,104 |
| ProQuest Research Library | ab(India OR "West Bengal") AND ab(policy OR policies OR scheme* OR program* OR intervention*) AND ab(drown* OR "near-drowning" OR water* OR safe* OR educat* OR rural OR child* OR infant OR infants OR swim* OR rescu* OR disaster* OR research OR submerge OR submerse OR injuries OR injury) | 25,261 |
| Bibliography of Asian Studies | (India* OR “West Bengal”) AND (policy OR policies OR scheme* OR program* OR intervention) AND (drown* OR “near-drowning” OR water* OR safe* OR educat* OR rural OR child* OR infant* OR swim* OR rescu* OR disaster* OR research OR submer* OR injur*) | 1,404 |
| Women’s Studies International | (India OR Indian OR “West Bengal”) AND (policy OR policies OR scheme* OR program* OR intervention) AND (drown* OR “near-drowning” OR water* OR safe* OR educat* OR rural OR child* OR infant* OR swim* OR rescu* OR disaster* OR research OR submer* OR injur*) | 773 |
